# Beyond minutiae: inferring missing details from global structure in fingerprints

**DOI:** 10.1186/s41235-025-00610-z

**Published:** 2025-02-04

**Authors:** Rachel A. Searston, Matthew B. Thompson, Samuel G. Robson, Jason M. Tangen

**Affiliations:** 1 School of Psychology, The University of Adelaide, Adelaide, Australia; 2 School of Psychology, Murdoch University, Perth, Australia; 3 Centre for Biosecurity and One Health, Harry Butler Institute, Murdoch University, Perth, Australia; 4 School of Psychology, The University of New South Wales, Sydney, Australia; 5 School of Psychology, The University of Queensland, Brisbane, Australia

**Keywords:** Natural image perception, Fingerprints, Forensic science, Expertise, Perceptual expertise, Visual inference, Fingerprint examination, Perceptual expertise, Forensic science, Gist perception, Scene-based recognition

## Abstract

Visual inference involves using prior knowledge and contextual cues to make educated guesses about incomplete or ambiguous information. This study explores the role of visual inference as a function of expertise in the context of fingerprint examination, where professional examiners need to determine whether two fingerprints were left by the same person, or not, often based on limited or impoverished visual information. We compare expert and novice performance on two tasks: inferring the missing details of a print at an artificial blank spot (Experiment 1) and identifying the missing surrounds of a print given only a small fragment of visual detail (Experiment 2). We hypothesized that experts would demonstrate superior performance by leveraging their extensive experience with global fingerprint patterns. Consistent with our predictions, we found that while both experts and novices performed above chance, experts consistently outperformed novices. These findings suggest that expertise in fingerprint examination involves a heightened sensitivity to gist, or global image properties within a print, enabling experts to make more accurate inferences about missing details. These results align with prior research on perceptual expertise in other expert domains, such as radiology, and extend our understanding of scene and face recognition to fingerprint examination. Our findings show that expertise emerges from an ability to combine local and global visual information—experts skillfully process both the fine details and overall patterns in fingerprints. This research provides insight into how perceptual expertise supports accurate visual discrimination in a high-stakes, real-world task with broader implications for theoretical models of visual cognition.

## Introduction

The sweeping outline of a scene catches our eye before we notice the intricate details within it—much like recognizing a friend from afar before discerning their facial features up close. Navon (1977) demonstrated this global precedence experimentally by showing participants a series of large letters composed of smaller ones. Participants identified the large letters more quickly than the smaller ones. Our capacity to rapidly process the ‘gist’ of complex images has since been widely demonstrated. Oliva and Torralba (2006) showed that people can quickly grasp the gist of a scene—distinguishing a bustling cityscape from a serene forest—by relying on low-dimensional spatial configurations that form a global summary of the whole image. Indeed, people can categorize natural scenes with remarkable accuracy at image resolutions as low as 32 × 32 pixels (Torralba, 2009; Wolfe & Kuzmova, 2011) and they can discriminate them at resolutions as low as 2 × 2 pixels (Searston et al., 2019). At these low image resolutions, the finer details vanish, leaving behind a global summary of the image as the basis for category judgments.

This ability to glean the global structure of a visual scene from low-dimensional information allows for rapid and accurate categorization, freeing up cognitive resources for detailed analysis of finer elements within the scene. The ability to rapidly extract global information and make accurate inferences based on limited visual input is a hallmark of human visual cognition (Brady, Stormer, & Alvarez, 2016; Oliva & Torralba, 2006). This rapid processing of global information is not only efficient but also serves to guide subsequent attention to relevant local features (Wolfe et al., 2011).

The processing of global and local information can also be likened to holistic and part-based mechanisms in face recognition. While global processing prioritizes an overarching summary of the visual input, akin to holistic processing, local processing focuses on analyzing finer, more granular features, akin to part-based strategies. Indeed, research in face recognition suggests that both holistic and part-based processing may contribute to superior visual recognition (Belanova et al., 2021). However, recent findings suggest that the contribution of holistic and part-based processing may differ across tasks and stages of processing. For example, while holistic processing is generally more dominant during recognition, part-based processing may play a crucial role during learning, particularly for unfamiliar faces (Leong, Estudillo, & Ismail, 2023; Chua & Gauthier, 2020). Additionally, individual differences in recognition ability are linked to both mechanisms, but not uniformly: some individuals rely more heavily on holistic processing, while others demonstrate superior featural analysis, reflecting distinct underlying strategies rather than a single holistic mechanism (Rezlescu et al., 2017). These findings show that global/holistic and local/part-based processing each contribute differently depending on the context—including the type of task, familiarity with the stimuli, and individual expertise.

Our capacity to extract global visual structure is critical not just for recognizing faces or categorizing natural scenes, but also for expert decision-making in domains like radiology and fingerprint examination. Radiologists can swiftly diagnose abnormalities in medical images at a momentary glance (Brennan et al., 2018; Nodine et al., 1999). Expert chess players can rapidly extract meaningful patterns from complex board configurations (Gobet & Simon, 1996; Palmeri, Wong, & Gauthier, 2004). Good tennis players can anticipate opponents’ movements well before they occur (Williams et al., 2002). And seasoned birdwatchers can efficiently identify different species in less than half a second (Tanaka & Curran, 2001). Some of these expert abilities are said to rely on holistic processing, where the configuration of features is processed as an integrated whole rather than as isolated parts (Tanaka & Farah, 1993; Gauthier & Tarr, 2002). Across domains, these feats of expertise demonstrate that a well-developed sensitivity to the global structure of a scene or an image is crucial for supporting accurate visual inferences in a variety of contexts. In the present study, we extend this work into the domain of fingerprint examination. We explore the role of global processing as a function of expertise by investigating the extent to which novices and experts can accurately distinguish fingerprints without the minutiae.

### Expertise in fingerprint examination

While media portray fingerprint examination as computer-driven, it fundamentally relies on human expertise. Expert examiners manually compare latent fingerprints found at crime scenes to prints in police databases. This comparison process is complicated by distortions and variations in latent impressions and the increasing similarity of prints retrieved by database searches as computer algorithms improve (Dror & Mnookin, 2010), and the potential for contextual information to introduce bias in expert judgments (Kukucka & Dror, 2023). The diverse range of cases means that examiners rarely build familiarity with any one individual’s prints. Despite these challenges, fingerprint experts exhibit remarkable accuracy in their comparison decisions, even under less-than-ideal conditions (Growns et al., 2023; Tangen et al., 2011; Tangen et al., 2020; Ulery et al., 2011).

The primary task of a fingerprint examiner is to infer whether two prints belong to the same finger or different fingers. This task is often described as a careful comparison of local features in the prints called ‘minutiae’—and experts outperform novices at searching and locating specific features in prints (Hicklin et al., 2019; Robson et al., 2021). In contrast to expectations from face recognition research, Vogelsang, Palmeri, and Busey (2017) found only weak evidence for holistic processing by experts using a composite fingerprint task adapted from the face recognition literature which suggests that local processing strategies may play a large role. However, experts can also reliably distinguish prints even when the minutiae are obscured or no longer available. Fingerprint experts can accurately identify prints clouded in visual noise (Thompson & Tangen, 2014) and presented after a time delay (Corbett et al., 2024). Studies using eye-tracking methods have shown that experts make smaller, more precise eye movements when viewing prints compared to novices (Busey & Vanderkolk, 2005; Busey et al., 2011), suggesting they are more adept at extracting global ‘holistic’ information without exhaustively searching for local features across various regions of a print (Busey & Parada, 2010).

Evidence suggests that fingerprint experts are sensitive to the global information distributed across different fingers of the same individual. Normally, these experts compare prints at the level of the individual *finger*: their task is to distinguish different impressions left by the same finger of the same individual (e.g., Smith’s right thumb) and different impressions left by different individuals. Searston and Tangen (2017c), however, tested whether fingerprint experts can also discriminate prints at the individual *person* level. In other words, how well can these experts distinguish between different impressions left by different fingers of the same person (e.g., prints from Smith’s right thumb, index, ring, middle or little fingers) and different impressions left by different fingers of different people. In this task, it is impossible to rely on a careful comparison of minutiae in each print because these local features and patterns vary across an individual’s fingers. Despite this variability, even novices performed above chance at distinguishing prints that were different impressions from different fingers of the same individual—and the experts were considerably more accurate than the novices. This example illustrates that there is also global structure distributed across an individual’s fingerprints and that experts have a heightened sensitivity to this global information relative to novices.

In casework, fingerprint experts are trained to conduct a detailed analysis of the minutiae in the latent print before comparing it to prints from known individuals (Robson et al., 2020, 2021), with some employing bias-reduction techniques like linear sequential unmasking to enhance decision accuracy (Dror et al., 2015). However, the above demonstrations suggest that fingerprint experts are not merely relying on local feature comparisons but are leveraging both global and local processing to achieve their remarkable accuracy. This sensitivity to global structure in prints likely comes about with extensive exposure to prints (Kellman & Garrigan, 2009; Richler & Palmeri, 2014). Longitudinal evidence shows that fingerprint trainees get better at discriminating fingerprints and fingerprint patterns as they progress through their on-the-job training to become experts (Searston & Tangen, 2017b, 2017c). More recent experimental evidence has also shown that statistical summary information can facilitate perceptual learning in fingerprint examination (Growns et al., 2022, 2023). This research suggests that experts are drawing on a mental repository of similar prints (Brooks, 1978; Medin & Schaffer, 1978) that allows them to build a richer global representation prior to feature segmentation (Oliva & Torralba, 2006)—and that this enriched global impression supports more efficient analysis of the finer details.

### Inferring missing details in fingerprints

A heightened sensitivity to global information may also facilitate accurate inferences based on incomplete data. Training to infer missing features from a category instance can result in better transfer to novel situations compared with standard training methods (Jones & Ross, 2011). Deducing that a bee with pale opalescent blue stripes on its abdomen must have a burrow made of soft stone is an inference that emphasizes commonalities *among* category members. Conversely, inferring the category label “blue banded bee” from the exemplar emphasizes information distinguishing *between* categories (Chin-Parker & Ross, 2004). This sensitivity to visual structure helps experts make accurate inferences even with imperfect visual information—crucial in the context of fingerprint examination.

Given the varied conditions under which fingerprint examiners work, they often need to make decisions based on incomplete information. Fingerprint experts sometimes work with pristine, fully rolled prints captured by a computerized fingerprint scanner. At other times, prints can be highly distorted or incomplete. Variation in surface, pressure, movement, skin residue, and even the compounds used to lift or capture a crime-scene (latent) print—such as phosphorescent dye—can affect how a print appears and what aspects of it might be missing. Imagine cradling a glass in your hand and loosening and tightening your grip. If you were to try this exercise, you may notice how different parts of each finger make contact with the glass, and that as you adjust your grip, your skin spreads and folds across the surface. An examiner’s appreciation for the gist of a print, and the redundancies dispersed across it, might help them infer what might be missing in these challenging circumstances.

### The present experiments

**Fig. 1:**
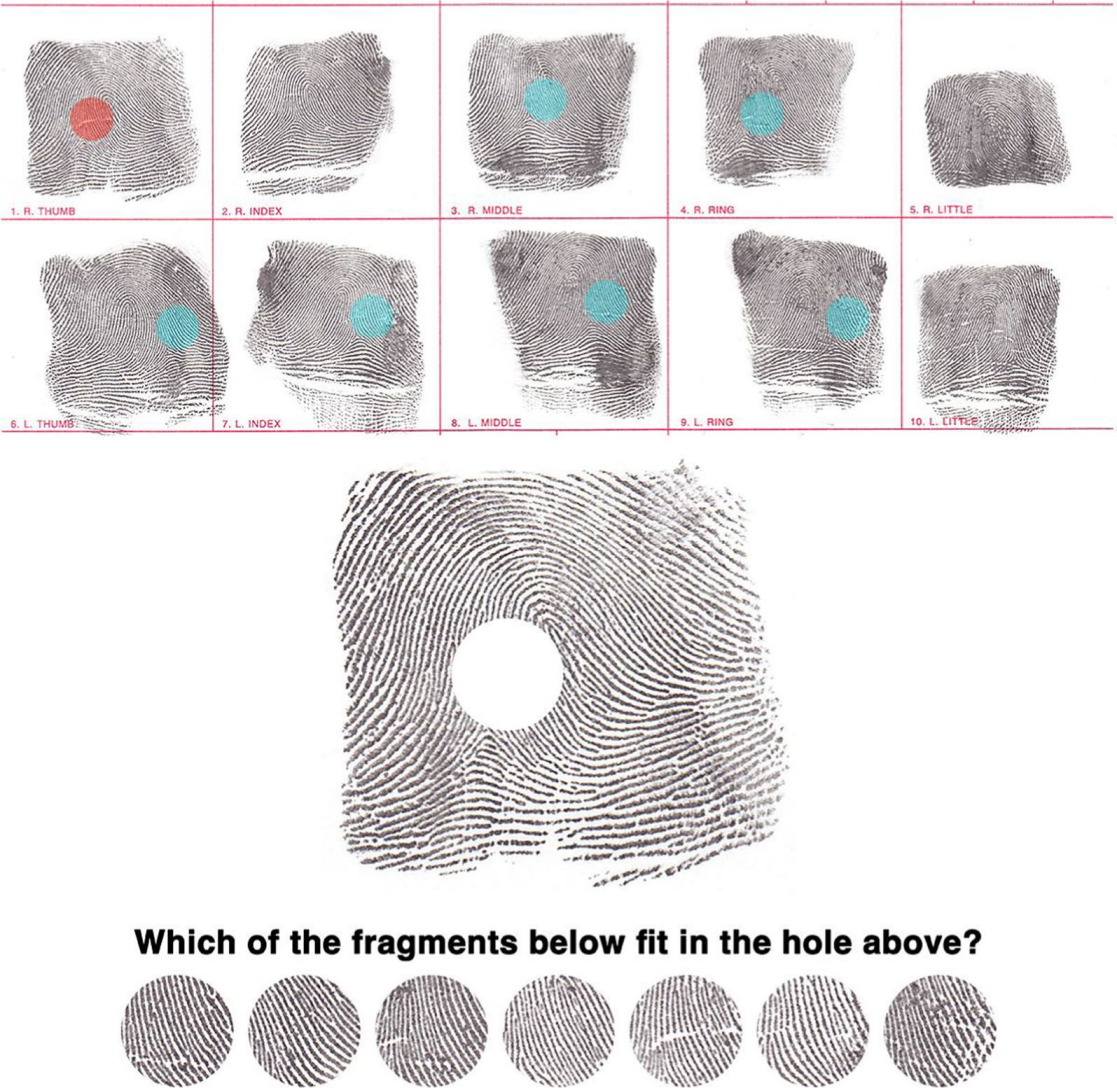
Fill-in-the-Fragment Task. The top panel shows ten fingerprints from a single individual (two hands, five fingers each) with circular patches highlighted in orange (target) or cyan (distractors). The bottom panel illustrates a single trial of the task. Participants were presented with a fingerprint containing a circular blank patch and asked to select which of the seven fragments below correctly fills the hole. The fragments include one target (matching the blank area) and six distractors, all extracted from different fingerprints of the same individual

The present experiments test the hypothesis that fingerprint experts can leverage global information to infer missing details in highly distorted or incomplete latent prints more effectively than novices. We designed two experiments to limit participants’ reliance on minutiae when comparing prints:

1. In Experiment 1, participants engage in a Fill-in-the-Fragment task (Fig. 1). They must infer the visual detail missing from a blank space cropped from a print, relying solely on the surrounding visual context of the print. This setup assesses their ability to use global context to reconstruct incomplete prints.
2. Experiment 2 employs a Fragment Comparison task. Participants compare small windows or ‘fragments’ of visual detail sampled from different regions (of different impressions) of the same finger, or different regions of a different finger altogether. Here, they must infer the missing visual surrounds of each fragment, further testing their capacity to use global visual patterns for accurate decisions.

By comparing the performance of experts and novices across these visual inference tasks, this research aims to understand how fingerprint experts use global information to make accurate decisions. We aim to determine whether their expertise enables them to compensate for missing or obscured minutiae by relying on global visual patterns. This investigation builds on previous studies exploring the role of global or holistic processing in face and scene recognition and seeks to isolate the role of global information in fingerprint examination.

### Experiment 1: Fill in the Fragment

In Experiment 1, we tested how well people can infer missing sections from a fingerprint and compared the performance of expert fingerprint examiners to that of novices. Building on Searston and Tangen’s (2017c) findings—which demonstrated that fingerprint experts can extract global information distributed across different fingers of the same person—we explored whether sensitivity to such distributed information would enable experts to infer missing visual details from a print. Specifically, we examined whether individuals could deduce the correct section of ridge detail missing from a print based on surrounding information—ridge flow, thickness, and friction ridge characteristics dispersed across a fingerprint.

To investigate this, we recruited a group of fingerprint experts and an age- and gender-matched group of fingerprint novices to complete a Fill-in-the-Fragment task. Participants had to infer the missing fragment of a fingerprint based on the surrounding context. The critical question was: can people use the global structure or style of a person’s fingerprint to accurately infer a small piece of missing friction ridge skin detail?

## Method

### Participants

#### Sensitivity analysis

We conducted a sensitivity analysis based on an estimated sample of 30 experts and 30 novices. Thirty matched expert-novice pairs, each completing 48 trials (totaling 2,880 observations), provided sufficient power (1 − *β* = 0.82) to detect a moderate difference between experts and novices (Cohen’s *d* = 0.45). We planned to collect data from as many experts as possible and then test an equal number of novices—as it is often difficult to recruit experts due to their busy schedules.

#### Expert group

We collected data from 44 expert fingerprint examiners (25 females, 19 males; median age = 42; min = 29; max = 60) from Australian state and federal police agencies. All experts were qualified, court-practicing fingerprint examiners. These experts completed the two tasks reported in this paper—along with seven other unrelated experimental tasks—in a random order over one or two days during breaks in their casework. The other tasks were unrelated to the research question addressed in this manuscript and have or will be reported elsewhere (e.g., Corbett et al., 2024; Robson et al., 2021, 2024). The examiners had an average of 15 years of experience examining fingerprints (min = 5, max = 40).

#### Novice group

Novice participants—with no formal experience in fingerprint examination—were recruited from The University of Adelaide, The University of Queensland, and Murdoch University communities. Forty-four novices (25 females, 19 males; median age = 43; min = 26; max = 62) participated for cash payment (AUD$20) and were ‘yoked’ or matched to experts based on age (± 2 years), gender, and level of education. Each expert was paired with a novice counterpart who had the same age, gender, and level of education. Additionally, the novice participants were incentivized to perform to the best of their ability by offered an additional cash payment (AUD$10) if they could exceed the performance of their expert counterpart.

#### Design

##### Task

In the ‘Fill-in-the-Fragment’ task, participants were presented with a fingerprint in the center of a computer screen containing a 132 × 132 pixel blank spot (see Fig. 1). The aim was to identify the fragment that correctly filled this blank spot from seven fragments displayed at the bottom of the screen. Each trial included one target fragment that corresponded with the blank spot and six distractor fragments from different fingerprints. The target fragment was randomly positioned on each trial—with a long-run probability of 1 in 7 (0.143) for guessing correctly.

##### Pilot

We chose seven fragments per trial to maximize variance between novices and experts. This decision was based on a pilot experiment with novices (*N* = 8) in which we tested the difficulty of the task with three, five, seven, or nine fragments. Novices correctly selected the target 73% of the time with three options, 59% with five options, 45% with seven options, and 42% with nine options. The task proved challenging with seven or more fragments—as novices made errors on more than half of the trials.

##### Trial sequencing

Each participant completed 48 unique trials, each featuring a new fingerprint and corresponding fragments. Novices were presented with the same trial sequences as their expert counterpart, ensuring identical stimuli and order for both groups. This matched-pairs design and method of yoking trial sequences between experts and novices ensures that any observed differences were most likely due to genuine differences in performance rather than variations in the stimuli.

##### Stimuli

All fingerprints were sourced from the National Institute of Science and Technology (NIST) Special Database 300 ‘rolled’ set (Fiumara, 1993). This set—originally donated by the United States Federal Bureau of Investigation—contains 8,871 prints collected in operational policing contexts, preserving their natural variation in quality, completeness, and contextual detail. For this experiment, we used a subset of 1,200 prints, including 10 prints of each finger type (e.g., thumb, index, middle, ring, and little fingers from both hands) from 120 individuals.

We standardized the width of all prints to 640 pixels while allowing the height to vary naturally, preserving the original aspect ratio of each print. From each of these standardized prints, a 132 × 132 pixel circular patch of friction ridge detail was removed, creating a set of 1,200 prints with missing fragments and 1,200 corresponding fingerprint fragments for targets and distractors. This fragment size represents approximately 4.25% of the total area of a print with dimensions of 640 × 640 pixels. All other original details in the prints—including natural variation in contrast, hue, and luminance—were left intact.

To ensure the task presented a challenge to participants, distractor fragments were extracted from different fingers of the same person, ensuring they were highly similar in overall pattern but different in detail to the target (see Searston & Tangen, 2017b). Each trial involved 48 fingerprints and their corresponding fragments, randomly sampled from one of 120 people. The fingerprint and target fragment were randomly selected from one of the person’s ten finger types (thumb, index, middle, ring, or little finger from either hand), while the distractor fragments were randomly selected from six of the remaining nine finger types.

The location of the missing fragment in the print varied from trial to trial, but fragments were systematically extracted by eye from similar parts of the finger to maximize target-distractor similarity on any given trial. For instance, if the target fragment was taken from the top left part of Smith’s left thumb, the distractors were taken from the top left parts of Smith’s other fingers that most closely resembled the target. This procedure ensured that distinctive minutiae between individual prints could not be used to distinguish the fragments.

##### Procedure

The task was presented to participants on a 13-inch MacBook computer. Participants first watched an instructional video explaining the task, including examples (see instructional video < https://youtu.be/YpStL-dAtS0 >). Following this, they viewed a total of 48 prints with blank spots, one at a time in sequence. Each trial displayed seven corresponding fragments (one target and six distractors) lined up below the fingerprint. Participants made their choice by clicking on the fragment they believed filled in the blank or missing detail in the print. Immediate feedback was provided—an audible tone and a green checkmark for correct answers, or a red “✕” for incorrect answers. The fingerprint and fragments remained on screen until the participant clicked on one of the seven fragments and during the 500-ms feedback window. There was a 500-ms interval between their response and the next trial. If participants took longer than 15 s to respond, a text prompt appeared during the inter-trial interval, stating: “Please try to make your choice in less than 15 s.” We allowed participants’ response times to vary naturally within this deadline to explore the dynamics of the decision-making process.

##### Hypotheses

Humans have an exceptional ability to recognize complex scenes with minimal detail (Navon, 1977; Oliva & Torralba, 2006; Searston et al., 2019). Applying this research to the current fill-in-the-fragment task, we hypothesized that both novices and experts would be able to identify the missing fragment by comparing global image properties with above-chance accuracy. However, given extensive research demonstrating experts’ superior ability to discriminate prints compared to novices—even under conditions with limited time and information (e.g., Searston & Tangen, 2017a; Thompson & Tangen, 2014)—we expected that experts would outperform novices. That is, while novices were expected to perform above chance, the performance of experts was expected to be significantly higher due to their vast exposure to a wide variety of prints and how they tend to look and vary.

## Results

The full dataset and accompanying analysis script (an R Notebook) for this experiment are available on the Open Science Framework at: https://osf.io/ndxpc.

### Proportion correct

#### Experts relative to novices

**Fig. 2:**
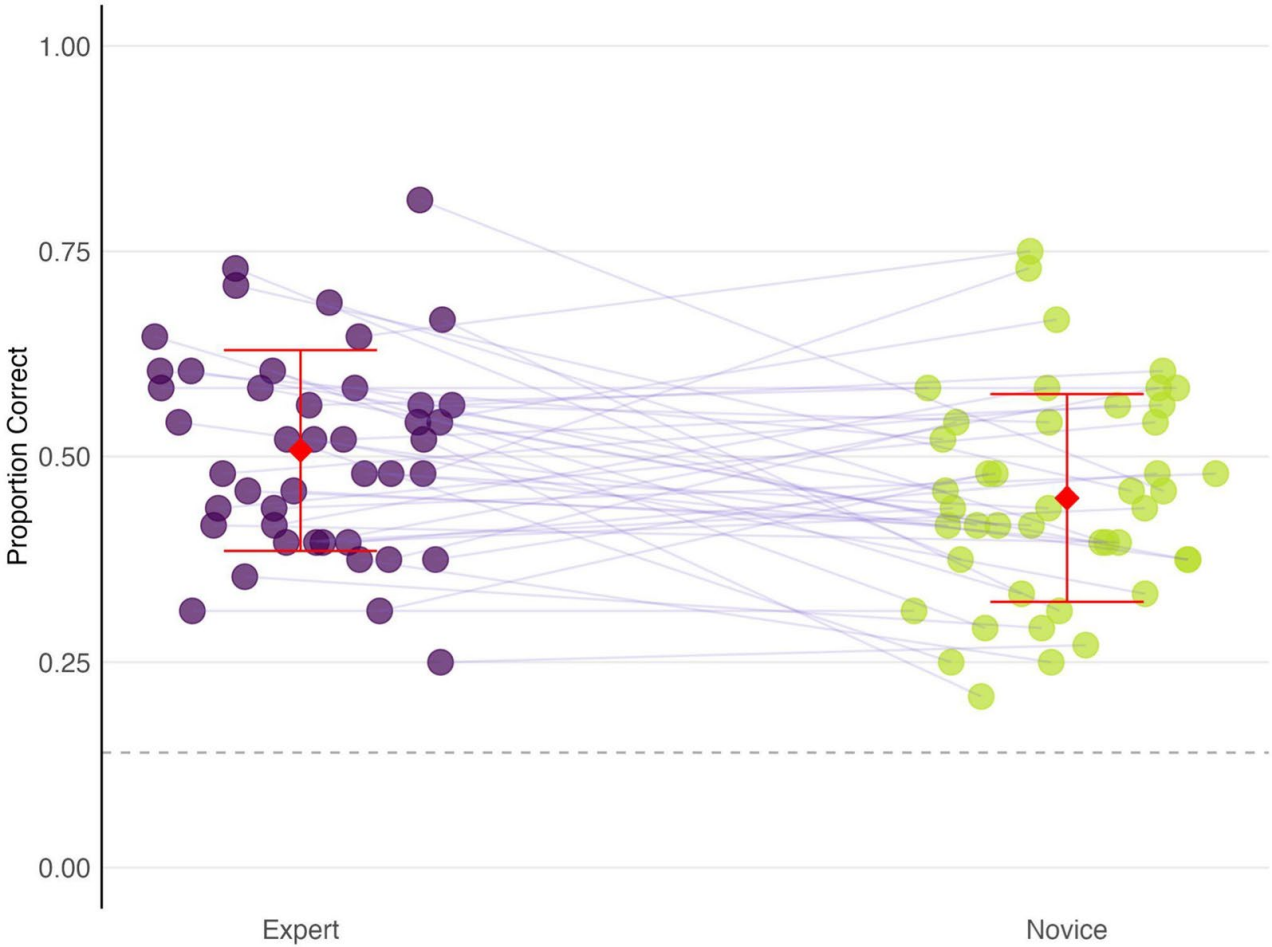
Proportion correct scores for expert and novice participants on the Fill-in-the-Blank task. Each point represents an individual participant’s performance. Red diamonds and error bars indicate the mean proportion correct and standard deviation for each group. Purple lines connect matched expert-novice pairs. Experts are coded as purple and novices as yellow-green. The dashed line indicates chance performance

Expert fingerprint examiners demonstrated a higher proportion of correct responses (*M* = 0.508, *SD* = 0.122) compared to novices (*M* = 0.450, *SD* = 0.126; see Fig. 2). A paired *t*-test confirmed this difference, *t*(43) = 2.295, *p* = 0.027—indicating that experts performed significantly better than novices. The mean difference was 0.058, with a 95% confidence interval ranging from 0.007 to 0.109, suggesting a moderate (Cohen’s *d* = 0.47) effect size.

#### Performance relative to chance

To further assess performance, we conducted one-sample t-tests comparing the proportion of correct responses of both experts and novices against the chance level of 0.143 (corresponding to a 1 in 7 probability of guessing the correct fragment). Expert performance was significantly above chance, with a mean proportion correct of 0.508 (*SD* = 0.122), *t*(43) = 19.988, *p* < 001. The 95% confidence interval for expert performance was between 0.470 and 0.545. Similarly, novice performance was also significantly above chance, with a mean proportion correct of 0.450 (*SD* = 0.126), *t*(43) = 16.280, *p* < 001. The 95% confidence interval for novice performance was between 0.411 and 0.488. These results indicate large effect sizes for both experts (Cohen’s *d* = 3.01) and novices (Cohen’s *d* = 2.45) when compared to chance.

### Response times

Response time analysis showed that experts had a mean response time of 11.49 s (*SD* = 4.19), while novices had a mean response time of 11.97 s (*SD* = 5.19). A paired *t*-test comparing the response times between experts and novices revealed no significant difference, *t*(43) = − 0.431, *p* = 0.668. The mean difference in response times was − 0.476 s, with a 95% confidence interval ranging from − 2.700 to 1.748 s. These results suggest that there was no significant difference in response times between experts and novices—indicating that both groups took a similar amount of time to respond on a given trial.

### Experiment 2: fragment comparison

In Experiment 1, participants could reliably identify missing sections of ridge detail in a fingerprint using the surrounding context of the print. Experts were also more accurate at identifying these missing fragments compared with novices. Since there was no overlapping local information between the fragments and the prints, these findings suggest that participants were using the surrounding visual context of the print to infer the missing local information. However, in this task, the fragments were extracted from the exact same image as the corresponding print. Since participants could mentally trace the ridges from the surrounding context to locate the target, above-chance performance could arise from processing local rather than global information. Experiment 2 addresses this limitation by testing whether experts and novices can use global information when local tracing is impossible.

In practice, fingerprint examiners do not ‘match’ images of prints per se; they distinguish between different instances or impressions made by the same finger and those made by different fingers. Fingerprint impressions made by the same finger can vary due to factors such as surface structure, perspiration, contaminants on the skin, skin flexibility, and pressure and movement during impression-making. Likewise, fingerprint impressions made by different fingers can look quite similar, due to the use of computer algorithms to speed up the search for comparison prints in police databases. As such, fingerprint experts do not match images, they match different impressions made by the same finger.

**Fig. 3:**
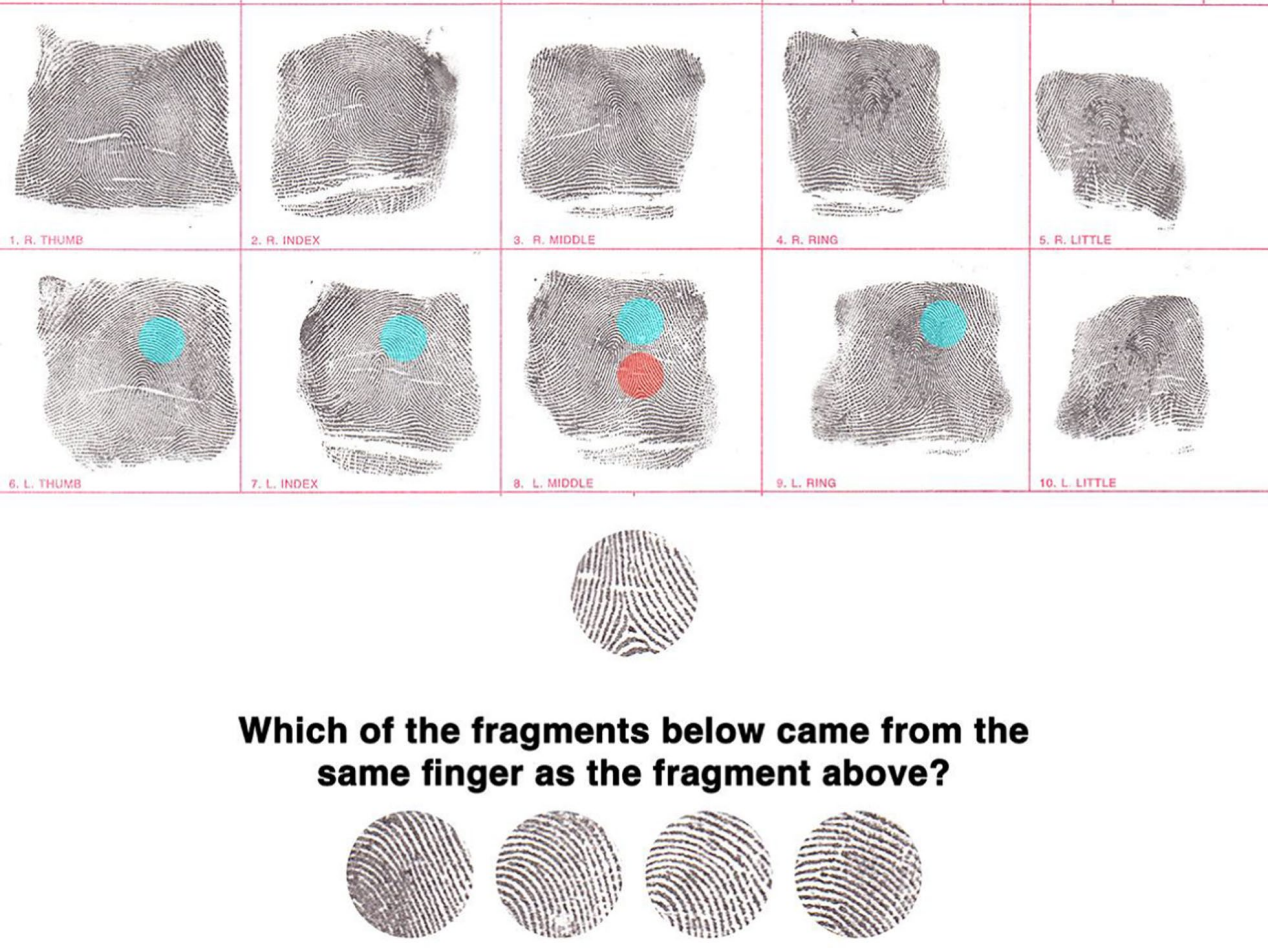
Fragment Comparison Task. The top panel displays fingerprints from all ten fingers of an individual (two hands, five fingers each). Circular patches highlighted in orange (target) and cyan (distractors) indicate potential sampling areas for fragments. Not depicted is that the probe and target fragments were always extracted from different impressions of the same finger (different tenprint cards), while distractors came from different fingers from different tenprint cards of the same individual. The bottom panel illustrates a single trial of the task. Participants were presented with a probe fragment (top) and asked to identify which of the four fragments below came from a different impression of the same finger. This task tests participants’ ability to discriminate fingerprints using global structural information without relying on specific overlapping minutiae

In Experiment 2, we tested whether participants could infer the identity of a fingerprint based solely on global information in a task where the corresponding fragments are taken from two different impressions of the same finger. Critically, these fragments were sampled from different local regions of the finger in each impression—such that they shared no overlapping features of friction ridge skin (see Fig. 3 for an example). Whereas participants in Experiment 1 inferred what fragment was missing given the surrounding visual context of a single impression, in Experiment 2 they needed to infer the surrounding visual context from the fragment of a *different* impression that was sampled from a different region of the finger. Mental tracing is impossible in this task for two reasons. First, the fragments come from different regions of the finger that share no overlapping ridge detail. Second, since the fragments are taken from different impressions at different times, local details can vary due to changes in pressure and other distortions during deposition.

Previous research has shown that fingerprint experts can discriminate same-source and different-source fingerprints with high accuracy (Tangen et al., 2011; Thompson et al., 2013a). In those studies, participants were able to compare overlapping features between two fingerprint impressions to decide if they were made by the same person or finger. In this current experiment, participants were given a small fragment of a fingerprint (132 × 132 pixels) and asked to identify which fragment—out of a lineup of four other fragments—came from the same finger. All but one of these four fragments were sampled from different regions of different fingers of the same individual. The corresponding fragment was sampled from a different impression and a different region of the same finger. We refer to this as the Fragment Comparison task.

We examined whether people could discriminate between two types of fragments: those sampled from different regions of different impressions of the same finger, and those sampled from different regions of different impressions of different fingers. This task forces participants to rely on just a small piece of friction ridge skin to infer the global structure or style of a person’s fingerprint.

## Method

### Participants, design and procedure

The same participants from Experiment 1—44 experts and 44 age- and gender-matched novices—completed the Fragment Comparison task in Experiment 2. The general procedure was identical to that of Experiment 1. Participants viewed an instructional video (see instructional video < https://youtu.be/HdFf2pzOR2Y >) before completing 48 trials of the Fragment Comparison task.

On each trial, a probe fragment from a new print was presented in the center of the computer screen (see Fig. 3). The probe was presented along with four other fragments at the bottom of the screen. One of these four fragments came from the same finger as the probe (“target”). The probe and the target fragments were extracted from different parts of different prints left by the same finger. The other three fragments in the lineup were from different fingers of the same individual (“distractors”). The target fragment was randomly positioned among the fragment lineup on each trial, and participants were asked to select the corresponding fragment each time. As in Experiment 1, corrective feedback and a prompt to respond within 15 s were provided on each trial with extended response times.

The distractors and targets were extracted from different fingers of the same person, and from the same part of the print on each trial. This procedure further increased the difficulty of the task—as the targets and distractors shared similarities based on the common visual structure present across an individual’s prints (e.g., see Searston & Tangen, 2017c). However, it also enabled us to isolate participants’ ability to identify individual fingers based on global image properties. We prepared trial sequences using different randomization seeds for each of the 44 expert-novice pairs, mirroring the same matched-pairs yoked sequence design as in Experiment 1. Each pair completed an identical trial sequence—ensuring they were perfectly matched on stimuli and order of presentation.

#### Stimuli

The materials for Experiment 2 were sourced from the NIST Special Database 300 ‘plain’ and ‘rolled’ sets (Fiumara, 1993). These sets include fingerprints taken from the same individuals at different times—encompassing 2 × fingerprints × 10 fingers from each donor. We selected four rolled prints and one plain print (or “slap”) from each of 200 donors, resulting in a total of 1,000 prints. The rolled prints were from four different fingers of the same individual donor, and the plain print was randomly chosen from one of these four fingers.

From each print, we extracted two fragments: one from the top half and one from the bottom half of the finger. This process yielded 1,600 rolled fragments for targets and distractors and 400 plain fragments for probes. Each fragment was manually cropped to a standardized size of 132 × 132 pixels to ensure that targets and distractors were selected from similar areas of the prints without overlapping with the probe.

The probe fragments were randomly chosen from either the top or bottom half of the plain prints. The four other fragments—including the target and three distractors—were sampled from the opposite part of the corresponding rolled prints from the same person. This method ensured that the probes were always taken from different parts of the finger than the target and distractor fragments. The friction ridge skin detailed in the probe fragment did not correspond with those in the target or distractors. Therefore, the probes and target fragments shared no specific minutiae in common. The question is whether the common global characteristics shared between the probe and the target fragments—such as general patterning, direction of ridge flow, ridge thickness, the individual’s general tendencies to apply more or less pressure—are sufficient for identifying prints left by the same finger.

#### Hypotheses

Building on the results of Experiment 1—where experts demonstrated superior ability to infer missing ridge details from fingerprints based on global image properties (e.g., ridge flow and patterning)—we hypothesized that in Experiment 2, both novices and experts would be able to identify matching fragments using similar global cues. However, we expected that experts would outperform novices due to their extensive experience with highly variable and impoverished latent impressions. Specifically, we predicted that novices would perform above chance, but experts would achieve significantly higher accuracy due to their exposure to fingerprint patterns and relationships between features.

## Results

The data analysis plan and workflow was the same as Experiment 1, and the data and script are available at: https://osf.io/ndxpc.

## Proportion correct

### Experts relative to novices

**Fig. 4:**
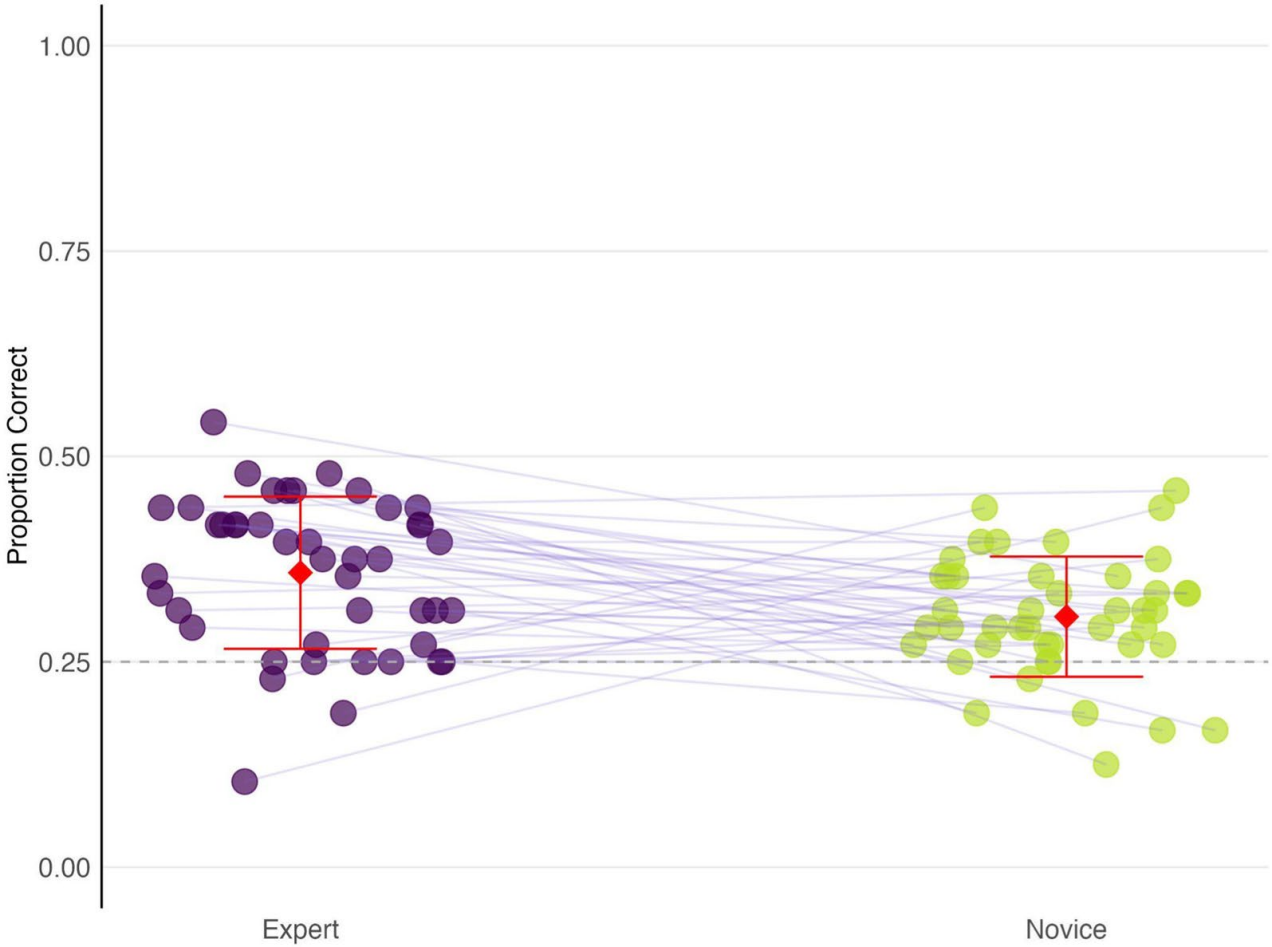
Proportion correct scores for expert and novice participants on the Fragment Comparison task. Each point represents an individual participant’s performance. Red diamonds and error bars indicate the mean proportion correct and standard deviation for each group. Purple lines connect matched expert-novice pairs. Experts are coded as purple and novices as yellow-green. The dashed line indicates chance performance

Expert fingerprint examiners demonstrated a higher proportion of correct responses (*M* = 0.358, *SD* = 0.093) compared to novices (*M* = 0.305, *SD* = 0.073; see Fig. 4). A paired *t*-test confirmed this difference, *t*(43) = 2.775, *p* = 0.008—indicating that experts performed significantly better than novices. The mean difference was 0.054, with a 95% confidence interval ranging from 0.015 to 0.092, suggesting a moderate effect size (Cohen’s *d* = 0.64).

### Performance relative to chance

As in Experiment 1, we conducted one-sample *t*-tests comparing the proportion of correct responses of both experts and novices against the chance level of 0.25 (corresponding to a 1 in 4 probability of guessing the correct fragment). Expert performance was significantly above chance, with a mean proportion correct of 0.358 (*SD* = 0.093), *t*(43) = 15.65, *p* < 0.001. The 95% confidence interval for expert performance was between 0.330 and 0.387. Similarly, novice performance was also significantly above chance, with a mean proportion correct of 0.305 (*SD* = 0.073), *t*(43) = 14.955, *p* < 0.001. The 95% confidence interval for novice performance was between 0.283 and 0.327. These results indicate large effect sizes for both experts (Cohen’s *d* = 2.36) and novices (Cohen’s *d* = 2.26) when compared to chance.

### Response times

Response time data showed that experts had a mean response time of 8.34 s (*SD* = 4.59) on the Fragment Task, while novices had a mean response time of 8.17 s (*SD* = 3.75). A paired t-test comparing the response times between experts and novices revealed no significant difference, *t*(43) = 0.173, *p* = 0.864. The mean difference in response times was 0.167 s, with a 95% confidence interval ranging from − 1.785 to 2.119 s. These results suggest that there was no significant difference in response times between experts and novices—indicating that both groups took a similar amount of time to respond on a given trial.

### General discussion

We conducted two experiments examining how fingerprint experts and novices use global visual information to make accurate inferences about missing details in fingerprints. Specifically, we aimed to determine whether experts, compared to novices, could more effectively use surrounding visual context to infer missing ridge detail in degraded or incomplete fingerprints. Our results show that while both groups can perform above chance, experts consistently outperform novices—demonstrating a heightened sensitivity to global image properties that enhances their ability to process incomplete or degraded prints.

In Experiment 1, participants could reliably identify missing sections of ridge detail by using the surrounding context, with experts showing significantly higher accuracy than novices. This supports the idea that experts have developed a refined sensitivity to the overall structure and pattern of fingerprints through extensive experience. This refined ability to quickly glean the gist of a print provides a foundation for accurate inferences about missing details. Previous research supports this interpretation, as experts in various domains demonstrate an ability to leverage global visual information for accurate and rapid decision-making (Brennan et al., 2018; Nodine et al., 1999; Oliva & Torralba, 2006). This ability to integrate global information aligns with mechanisms of holistic processing described in the face recognition literature, where the spatial configuration of features is perceived as an integrated whole, facilitating rapid and accurate decisions (Belanova et al., 2021; Chua & Gauthier, 2020).

Experiment 2 extended these findings by testing whether participants could discriminate fingerprint fragments sampled from *different* impressions of the same finger. Critically, these fragments were also sampled from different regions of the different impressions—such that they shared no overlapping local features of friction ridge skin, unlike a typical fingerprint matching task. Experts again outperformed novices, demonstrating their superior ability to use global image properties to draw inferences even when information is limited, and local minutiae are unavailable. This further supports the hypothesis that experts rely on a rich mental repository of fingerprint patterns, enabling them to build a global representation of a print that aids in accurate comparison decisions (Brooks, 1978; Medin & Schaffer, 1978).

While performance was significantly above chance in both experiments, it was generally quite poor relative to other fingerprint matching experiments (e.g., Thompson, et al., 2013b). This outcome is not surprising given the challenging nature of the task—the fragments were small (132 × 132 pixels), and the distractors were highly similar to the target fragments, all sampled from different regions of the same individual’s fingerprints. However, the generally poor performance indicates that more information and time to conduct a detailed analysis of minutiae is also critical to making accurate comparison decisions (Robson et al., 2021). These findings align with the idea that fingerprint experts heavily rely on local or part-based processing, particularly when local minutiae provide diagnostic features (Vogelsang, Palmeri, & Busey, 2017). However, the persistence of expert-novice differences in the absence of local diagnostic information suggests that experts may be able to switch between global (holistic) and local (part-based) mechanisms depending on the visual context of the case. Much like scene and face recognition, holistic impressions of fingerprints may guide attention to key local features, enabling experts to balance efficiency and accuracy in their interpretation of visual evidence.

In general, our findings complement previous research showing that perceptual expertise involves the ability to quickly and accurately process global visual information (Busey & Vanderkolk, 2005; Thompson & Tangen, 2014; Thompson et al., 2014). We also add to existing research on scene and face recognition by demonstrating that a capacity to extract global visual information can support accurate decision-making in a complex visual comparison task. Our findings show that this ability is not limited to natural scenes and faces but extends to the specialized expert domain of fingerprint examination—where examiners must often make decisions based on incomplete or degraded prints. This insight is relevant to a range of contexts where information is compromised, such as when radiologists detect abnormalities in low-resolution medical images (Boita et al., 2021), police identify suspects from blurry surveillance footage (Burton et al., 1999), or remote sensing experts interpret satellite imagery when images are affected by atmospheric interference or resolution limitations (Ahn et al., 2023). Future research may wish to explore the generality of our findings to such contexts.

The current experiments also extend on prior studies showing that fingerprint experts can infer the identity of a print by comparing impressions of different fingers from the same person (Searston & Tangen, 2017c). Perceptual expertise in fingerprint examination appears not to rely solely on detecting and comparing local features (Hicklin et al., 2019; Robson et al., 2020, 2021), but also on information distributed across a print and between different prints. This idea is similar to other empirical findings suggesting that expertise in fingerprint examination rests partly on sensitivity to global or holistic information (Busey & Vanderkolk, 2005; Busey & Parada, 2009; Thompson & Tangen, 2014). For example, Thompson and Tangen (2014) showed that fingerprint experts can accurately match prints clouded in noise or prints presented only very briefly. A careful comparison of local features cannot explain these expert-novice differences.

Moreover, our research suggests that while novices can perform above chance in these tasks, expertise substantially enhances the ability to use global visual information for accurate fingerprint comparison decisions. This highlights the importance of experience and extensive exposure to a wide variety of prints in developing the perceptual skills needed for expert performance (Searston & Tangen, 2017b, 2017c).

While our findings provide insight into the perceptual mechanisms underlying expertise in fingerprint examination, they should not be taken as evidence that examiners rely on inferred details in operational settings. They should also not be used as a validation of expert performance under challenging casework conditions in forensic reporting or court testimony. Instead, these results highlight how developing a sensitivity to global image properties might support fingerprint comparison decisions under controlled conditions, contributing to our general understanding of perceptual expertise. Future research should examine how global and local processing work together in expert decision-making, and test training methods that develop both abilities in novice analysts (see Growns et al., 2022; Robson et al., 2022; Searston et al., 2017b for examples of effective training). Additionally, examining how experts integrate global and local information under different conditions could provide deeper insights into the cognitive mechanisms underlying fingerprint expertise (Robson et al., 2021).

In conclusion, our study provides evidence that fingerprint expertise involves leveraging global visual information alongside local minutiae. Under controlled experimental conditions, experts demonstrated superior ability to leverage global properties of fingerprints—such as ridge flow patterns—to make accurate comparisons. This heightened sensitivity to global patterns may guide experts’ attention to relevant local features, enabling more efficient and accurate detailed analysis. Although these results advance our theoretical understanding of perceptual expertise, we emphasize that they should not necessarily be used to inform or validate operational fingerprint examination procedures. Rather, these findings further reveal the perceptual mechanisms that characterize expert performance in high-stakes visual comparison domains, from fingerprint examination to medical image interpretation. What emerges is a defining feature of perceptual expertise: the ability to rapidly process global visual information while maintaining precise attention to local detail.

## Data Availability

The data for each novice and expert participant, and the code used to produce our results and plots, are available on the Open Science Framework—with the exception of identifiable demographic information: https://osf.io/mw9ka. The preregistrations for Experiment 1 < https://osf.io/d2vqk > and Experiment 2 < https://osf.io/f9vkc > are also available on the Open Science Framework. We do not have permission to share the fingerprint materials used in these experiments; however, a copy of the complete original set can be requested at: https://www.nist.gov/itl/iad/image-group/nist-special-database-300.
